# Supplementary material for: Fetal brain response to maternal inflammation requires microglia
Source: Development. 2024 May 22;151(10):dev202252. doi: 10.1242/dev.202252 (PMC11190434; doi:10.1242/dev.202252)
Supplement: Supplementary information [file develop-151-202252-s1.pdf]

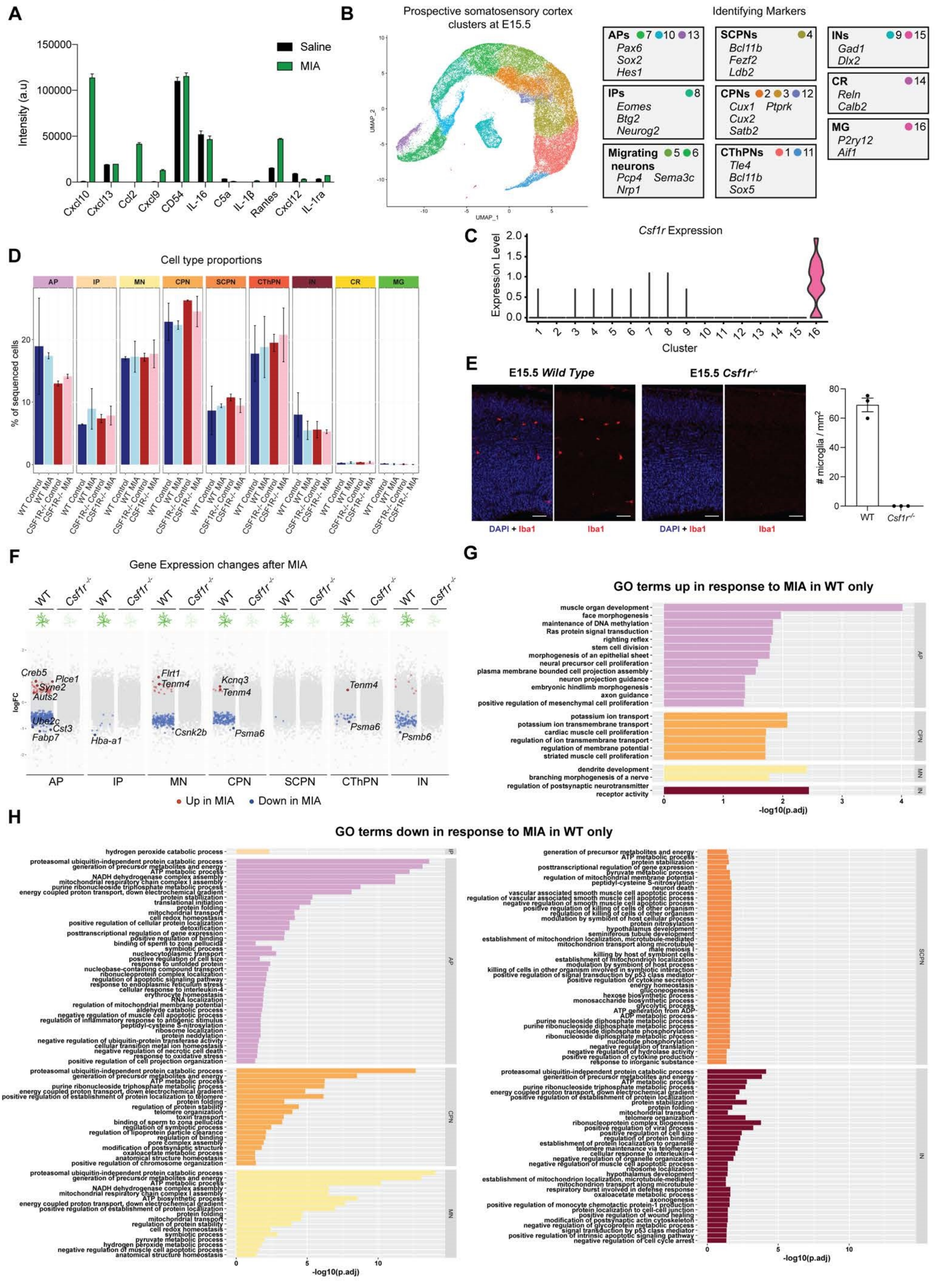

Development • Supplementary information

Fig. S1. Response to maternal immune activation.

(A) Bar plot showing cytokine expression analysis in spleens of pregnant dams 5h after being injected with saline or Poly(I:C) (MIA), measured via cytokine array profiling (see Supplementary Materials and Methods). (B) UMAP visualization of snRNAseq from the developing somatosensory cortex at E15.5. All samples from WT and *Csf1r* null, MIA and saline control-treated, are included. Cells are colored by cluster (left). Canonical marker genes used for cell type identification are shown on the right, along with the cluster numbers that correspond to each cell type. (C) Violin plot showing the expression of *Csf1r* in microglia (cluster 16) but no other cortical cell types. (D) Bar plot showing cell type proportions after maternal injection of saline as compared to Poly(I:C) in WT and *Csf1r* null mice. Generalized mixed effects binomial model showed no significant changes in substate proportions between treatment groups. Error bars represent 1 standard deviation. (E) Representative images and quantification of Iba1 immunostaining in *Wild Type* and *Csf1r*$^{-/-}$ prospective somatosensory cortex at E15.5 (scale bar 50μm). (F) Dot plot of log(fold change) in gene expression between MIA and saline for cell types with significant changes at E15.5. DEG analysis performed using DESeq2. AP, apical progenitors; IP, intermediate progenitors; MN, migrating neurons; CPN, callosal projection neurons; SCPN, subcerebral projection neurons; CThPN, corticothalamic projection neurons; IN, interneurons. (G) Enriched Gene Ontology (GO) terms from differentially expressed genes upregulated in response to MIA in WT but not in CSF1R KO mice. (H) Enriched GO terms from differentially expressed genes downregulated in response to MIA in WT but not CSF1R KO mice.

Development • Supplementary information

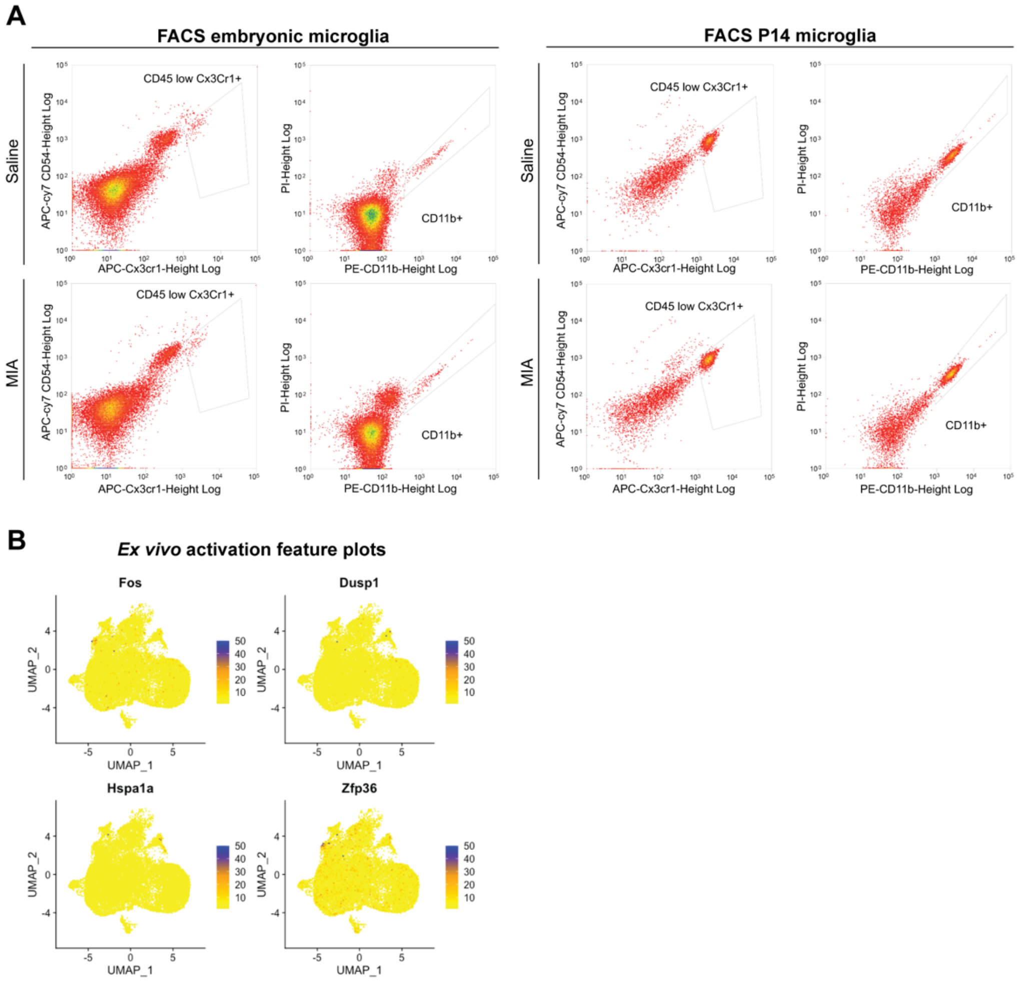

Fig. S2. Microglia isolation by FACS.

(A) FACS plots of microglia sorting in embryonic and postnatal day 14 (P14) mice after maternal injection of saline or Poly(I:C) (MIA). Prior to FACS purification, microglia were labeled with antibodies against CD45, CX3CR1 and CD11b. Boxes show gating for each parameter. (B) UMAP visualization of microglia scRNAseq data from the developing somatosensory cortex of WT mice. Plots are colored for expression of known microglia *ex vivo* activation markers.

Development • Supplementary information

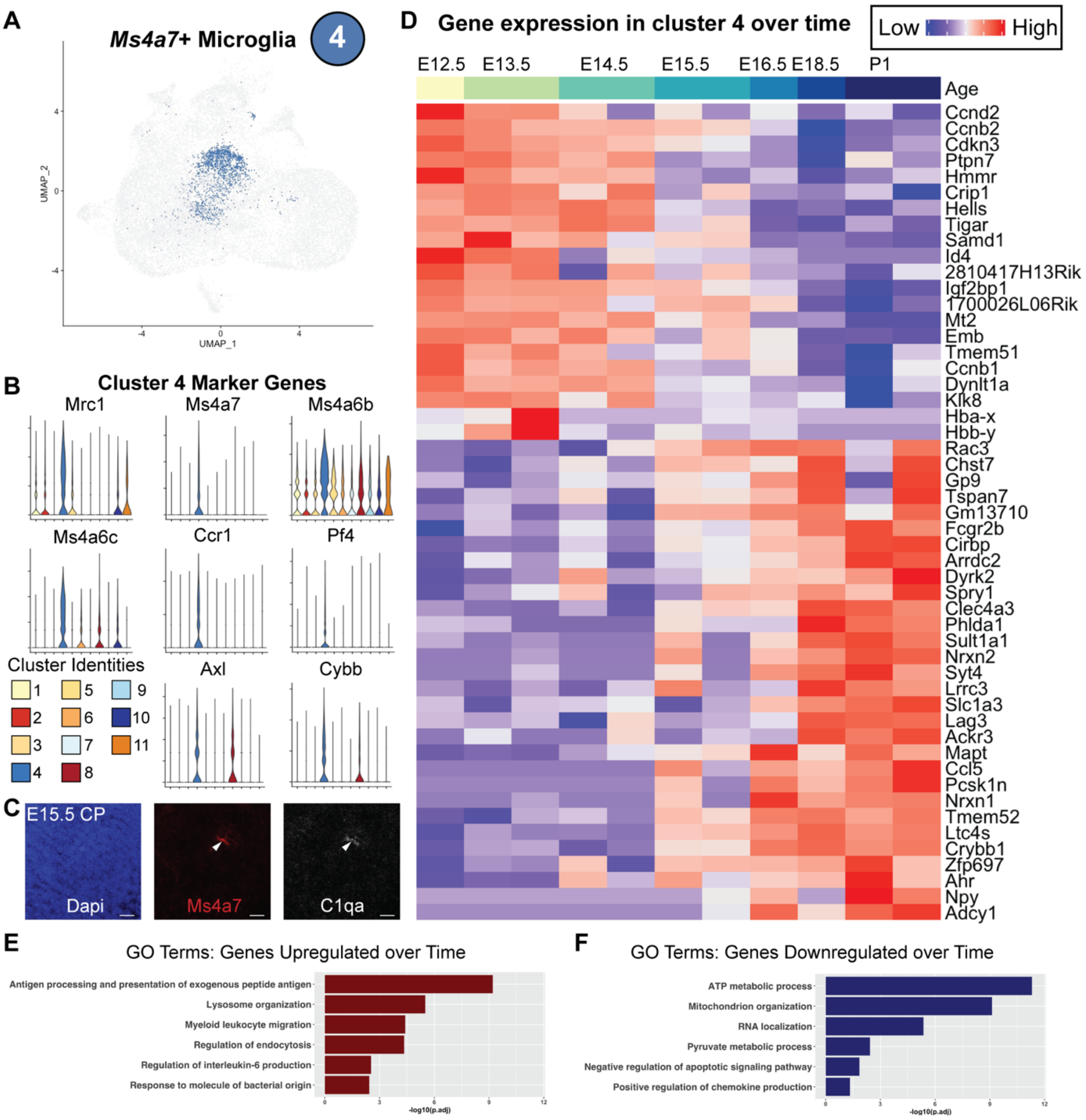

Fig. S3. Ms4a7+ microglia characterization.

(A) UMAP visualization of microglia scRNAseq data from the developing somatosensory cortex of WT mice. Cells belonging to microglia cluster 4 are colored. (B) Violin plots showing the expression of microglia cluster 4 marker genes in all clusters. (C) Fluorescent images from RNA *in situ hybridization* (RNAScope) showing colocalization of Ms4a7 and C1qa (pan-microglial marker) in the mouse cortical plate (CP) at E15.5 (scale bar: 20 μm). (D) Heat map of gene expression in cluster 4 over time. (E) Bar plots showing enriched Gene Ontology (GO) terms in genes upregulated over time in microglia cluster 4. (F) Bar plots showing enriched GO terms in genes downregulated over time in microglia cluster 4.

Development • Supplementary information

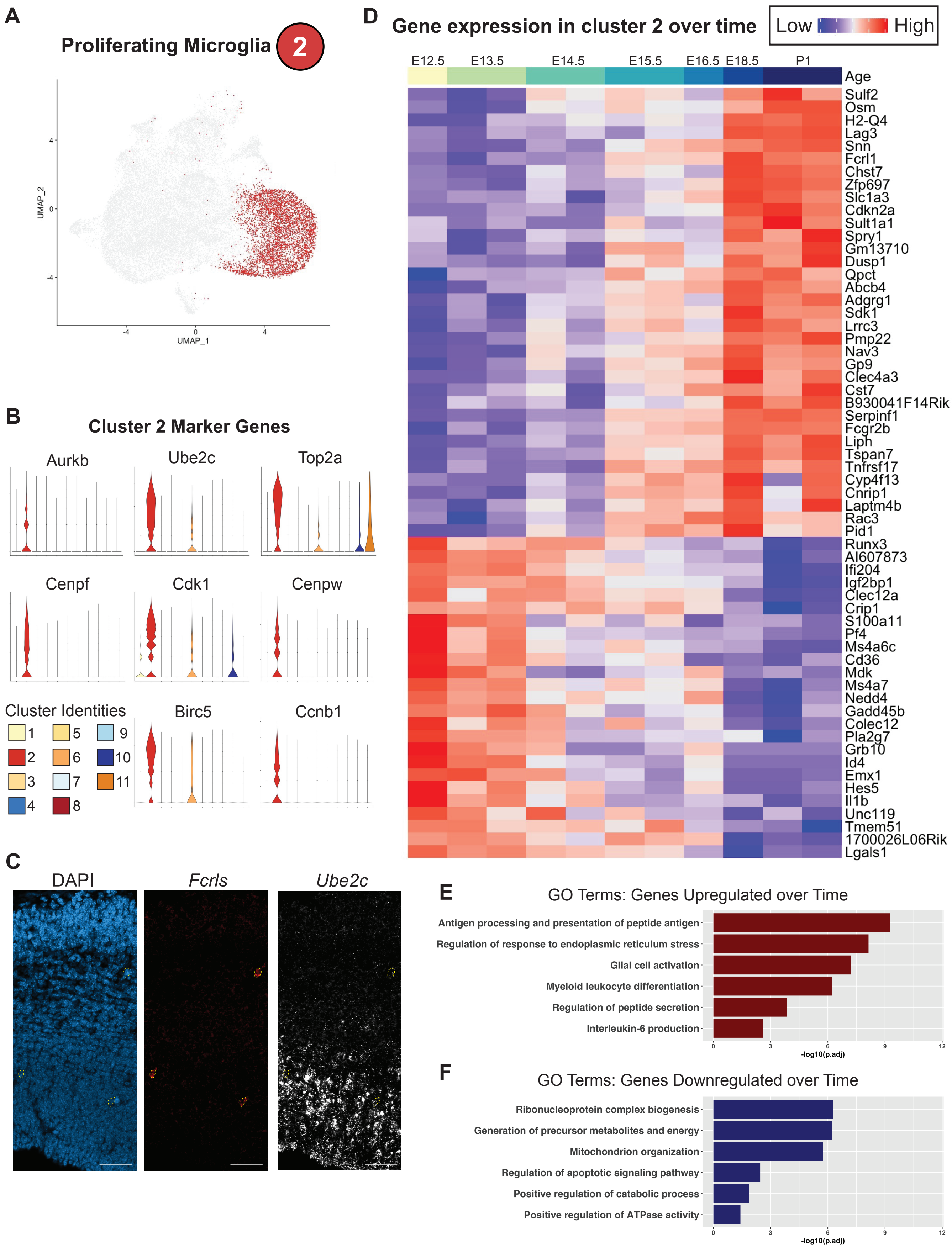

A
Proliferating Microglia
2
UMAP_2
UMAP_1
4
0
-4
B
Cluster 2 Marker Genes
Aurkb
Ube2c
Top2a
Cenpf
Cdk1
Cenpw
Cluster Identities
1
2
3
4
5
6
7
8
9
10
11
Birc5
Ccnb1
C
DAPI
Fcrls
Ube2c
D
Gene expression in cluster 2 over time
Low
High
E12.5
E13.5
E14.5
E15.5
E16.5
E18.5
P1
Age
Sulf2
Osm
H2-Q4
Lag3
Snn
Fcrl1
Chst7
Zfp697
Slc1a3
Cdkn2a
Sult1a1
Spry1
Gm13710
Dusp1
Qpct
Abcb4
Adgrg1
Sdk1
Lrrc3
Pmp22
Nav3
Gp9
Clec4a3
Cst7
B930041F14Rik
Serpinf1
Fcgr2b
Liph
Tspan7
Tnfrsf17
Cyp4f13
Cnrip1
Laptm4b
Rac3
Pid1
Runx3
AI607873
Ifi204
Igf2bp1
Clec12a
Crip1
S100a11
Pf4
Ms4a6c
Cd36
Mdk
Ms4a7
Nedd4
Gadd45b
Colec12
Pla2g7
Grb10
Id4
Emx1
Hes5
Il1b
Unc119
Tmem51
1700026L06Rik
Lgals1
E
GO Terms: Genes Upregulated over Time
Antigen processing and presentation of peptide antigen
Regulation of response to endoplasmic reticulum stress
Glial cell activation
Myeloid leukocyte differentiation
Regulation of peptide secretion
Interleukin-6 production
0
3
6
9
12
-log10(p.adj)
F
GO Terms: Genes Downregulated over Time
Ribonucleoprotein complex biogenesis
Generation of precursor metabolites and energy
Mitochondrion organization
Regulation of apoptotic signaling pathway
Positive regulation of catabolic process
Positive regulation of ATPase activity
0
3
6
9
12
-log10(p.adj)

Fig. S4. Proliferating microglia characterization.

(A) UMAP visualization of microglia scRNAseq data from the developing somatosensory cortex of WT mice. Cells belonging to microglia cluster 2 are colored. (B) Violin plots showing the expression of microglia cluster 2 marker genes in all clusters. (C) Fluorescent images from RNA *in situ hybridization* showing colocalization of Ube2c and Fcrls (pan-microglial marker) in the mouse cortex at E15.5 (scale bar: 50 μm). (D) Heat map of gene expression in cluster 2 over time. (E) Bar plots showing enriched Gene Ontology (GO) terms in genes upregulated over time in microglia cluster 4. (F) Bar plots showing enriched GO terms in genes downregulated over time in microglia cluster 4.

Development • Supplementary information

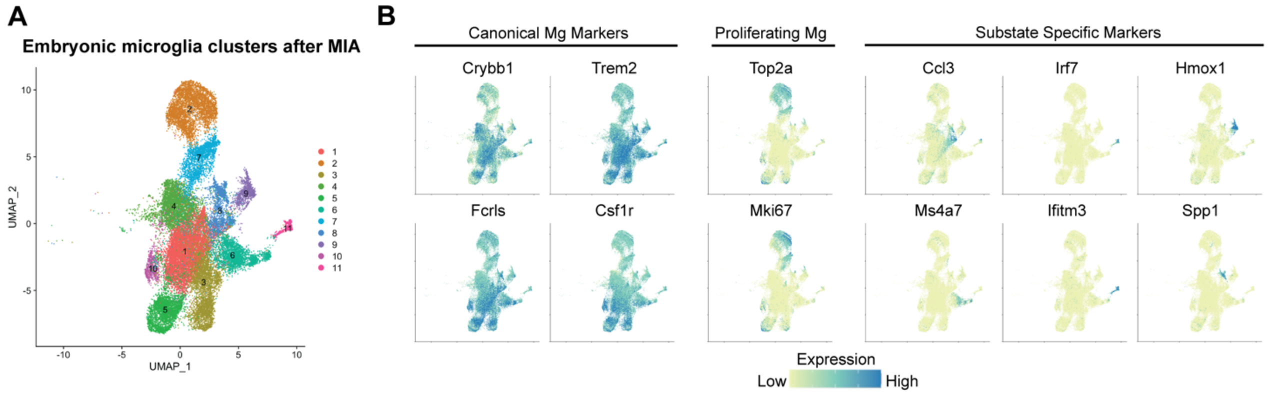

Fig. S5. Embryonic microglia clusters after MIA.

(A) UMAP visualization of microglia scRNAseq data from the developing somatosensory cortex of mice after maternal injection of Poly(I:C) (MIA). UMAP shows all cells from MIA and saline-injected animals analyzed at both E13.5 and E15.5. Cells are colored by cluster. (B) UMAP plots from A colored for expression of canonical, proliferating and substate specific microglia marker genes.

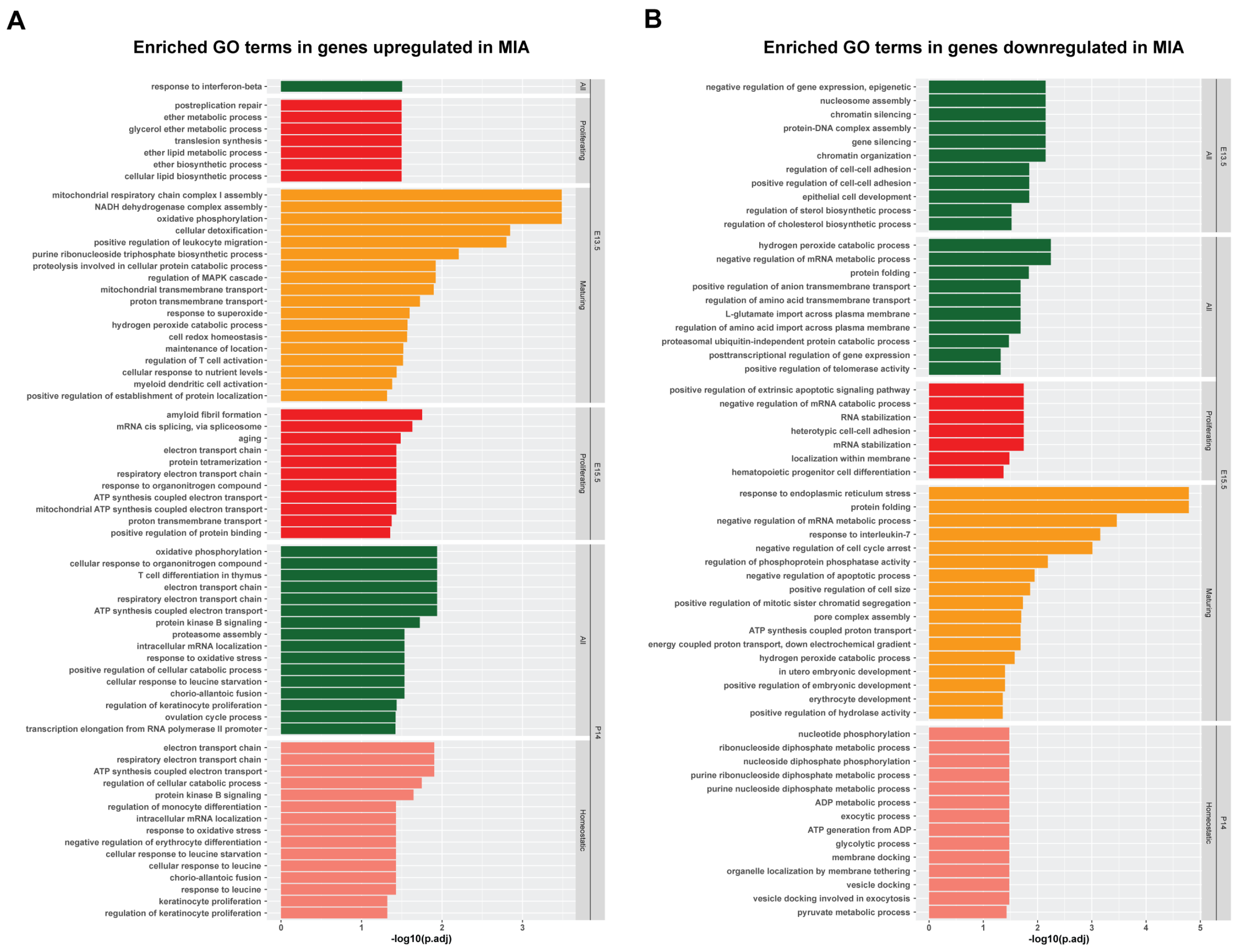

Fig. S6. Gene ontology analysis in embryonic and postnatal microglia after MIA.

(A) Bar plots showing enriched Gene Ontology (GO) terms from differentially expressed genes upregulated in response to MIA in WT mice. (B) Bar plots showing enriched GO terms from differentially expressed genes downregulated in response to MIA in WT mice.

Development • Supplementary information

SUPPLEMENTARY MATERIALS AND METHODS

Mouse husbandry

All animal procedures were approved by the Harvard University IACUC and conducted in compliance with institutional and federal guidelines. Mice were housed in individually ventilated cages using a 12-hour light/dark cycle and had access to water and food *ad libitum*. Males only were used for snRNA-seq experiments of cortex from *CSF1R* mutant versus WT mice, given the published male predominance of behavioral effects and changes in neuronal gene expression after MIA(Xuan and Hampson, 2014; Gogos *et al.*, 2020; Kalish *et al.*, 2021). Equal ratios of male and female mice were used in scRNA-seq of FACS-purified microglia in postnatal samples in which sex could be determined. We also used both female and male mice in scRNA-seq experiments of FACS-purified embryonic microglia but were unable to prospectively determine sex to verify equal ratios of males and females. This limitation existed because tissue was processed for single cell isolation and sequencing immediately following brain dissection to minimize *ex vivo* microglia activation, which did not allow time for genotyping between embryo dissection and sample preparation.

Maternal Immune Activation

C57Bl/6 WT timed pregnant female mice or CSF1R$^{+/-}$ female mice that had undergone timed mating with CSF1R$^{+/-}$ male mice were injected intraperitoneally at E12.5 with 20mg/kg Poly(I:C) (Sigma, P0913) resuspended in 0.9% sterile sodium chloride (Thermo Fisher Scientific NC9016275). Control animals were injected with an equal volume of 0.9% sodium chloride. Confirmation of the expected maternal inflammatory response using this protocol was done by cytokine analysis via a membrane-based immunoassay per manufacturer instructions (R&D Systems, ARY006), performed on maternal splenic tissue obtained after sacrificing animals not used for sequencing experiments 5 hours after Poly(I:C) injection. Cytokine levels were assessed using the Image Lab software (BioRad) and statistics performed with GraphPad Prism (t-test with Holm-Sidak multiple comparison correction). Mice were note disturbed after MIA until the time of euthanasia for RNA analysis.

RNA Fluorescent *in situ* Hybridization and Immunohistochemistry

Brains for ISH were prepared by transcardial perfusion of ice-cold Phosphate Buffered Saline (PBS), then ice-cold 4% paraformaldehyde (Electron Microscopy Sciences, 15710) diluted in PBS. Brains were stored overnight in 4% paraformaldehyde at 4°C, followed by sequential washes with PBS and gradually increasing concentrations of sucrose in PBS at 4°C for cryoprotection per the ACDBio Multiplex Fluorescent V2 Assay kit protocol (ACDBio, 323100). Brains were frozen in a solution of 2-parts 30% sucrose, 1-part OCT (Tissue-Tek, 4583) in Peel-A-Way embedding molds (VWR, 15160-215) and stored at -80°C. PFA-fixed tissues were cryosectioned at 16 um. For ISH tissue was heated at 70°C prior to use to adhere tissues to glass slides. Sections were hydrated with PBS for 5 minutes, dried and re-hydrated 5 minutes to remove residual OCT. Target retrieval solution (ACDBio 322000) was heated to a rolling boil and incubated on the slides for 5 minutes followed by two quick rinses of water and dehydration with 100% ethanol. Protease III reagent (ACDBio, 322340) was applied to the slides for 10-20 minutes at

Development • Supplementary information

40°C. Slides were washed 2x in water, followed by pooled probe hybridization and signal amplification according to manufacturer's protocols. RNAscope Catalog Target Probes used were: *Fcrls* (ACDBio, 441231-C2) *C1qa* (ACDBio, 441221-C3), *Ube2c* (ACDBio, 552191-C2), *Ms4a7* (ACDBio, 314601), *Hmox1* (ACDBio, 498811), *Irf7* (ACDBio, 534541-C3), *Fabp5* (ACDBio, 504331), *Spp1* (ACDBio, 435191), *Hes1* (ACDBio, 417701) and *Lars2* (ACDBio, 593351-C2). For immunohistochemistry, tissue was blocked and permeabilized for 2 hours at room temperature in blocking solution (PBS with 0.5% BSA, 0.3% Triton X-100, and 2% donkey serum), and incubated overnight at 4°C in blocking solution with rabbit anti-Iba1 (1:100, Wako, 019-19741). After washing of primary antibody, secondary antibody incubation was done for 2h at room temperature in blocking solution with donkey anti-rabbit IgG (H+L) Alexa Fluor 546 (1:500, Thermo Fisher, A10040).

Imaging and quantification

Confocal imaging of RNAscope *in situ* hybridization-stained tissue sections was performed using a Zeiss 700 or LSM900 inverted confocal microscope. Tile scan images spanning the width of the dorsal cortical wall, from pia to corpus callosum, were captured using a 20x air objective and stitched using Zeiss Zen Software (version 2.6). Z-stack images were acquired using the 20x or 63x oil-immersion objective with a 0.5 um Z-step. All confocal imaging was captured using Zen Black software (Zeiss, v2.3). Fluorescence imaging of Iba1 immunostaining was performed using a Zeiss Axio Imager 2 upright microscope with a 10x air objective using Zeiss Zen Software.

RNA scope and immunostaining images were quantified using Fiji by an investigator blinded to the experimental condition (Schindelin *et al.*, 2012). For RNAscope analysis, mRNA speckles per microglia were counted across the cortical plate. For Iba1 immunostaining, Iba1$^+$ cells were counted across the cortical wall (from pia to corpus callosum).

Tissue Dissection for RNA Sequencing

For experiments involving snRNA-seq or scRNA-seq of embryonic brain tissue, we euthanized the pregnant females at the age of analysis and obtained the embryos. For scRNA-seq of postnatal microglia, mice were housed with their mothers and littermates until euthanasia on the day of analysis. Brain dissection was performed with RNAse-free technique in ice cold Hybernate-E (Brainbits) for embryonic tissue or in ice cold Hibernate-A (Brainbits) for postnatal tissue. The somatosensory (S1) cortex (or prospective S1 cortex for embryonic tissue) was dissected and meninges removed. Tissue was immediately processed as described below for scRNA-seq experiments or flash frozen in liquid nitrogen and stored at -80°C until genotypic was completed for snRNA-seq experiments.

Single-cell Suspension Preparation and FACS

Live microglial suspensions from embryonic and postnatal dissected tissues were prepared for fluorescence activated cell sorting (FACS) and maintained under ice-cold conditions as previously described (Stogsdill *et al.*, 2022). Prospective S1 cortex blocks were minced and transferred to a 5 mL

Development • Supplementary information

microcentrifuge tube with Hibernate-A and pelleted at 500 *g* for 5 min, and then the supernatant was decanted. The pellet was resuspended in 1 mL dissociation buffer (20 mL PBS (Gibco, 15630-080) + 20 uL RNasin (Promega, 40 units/ul, N2515) + 400 uL DNAse (Worthington, 12,500 units/mL, LS002006)) and transferred to a 2 mL Dounce homogenizer (Sigma D8938-1SET). The tissue was homogenized with 15 plunges of pestle “A”. Homogenate was passed through a 70 um cell strainer (Miltenyi, 130-110-916), then pelleted at 500 *g* for 5 min. The pellet was resuspended in 500 uL FACS buffer (PBS with 0.04% Bovine Serum Albumin (BSA, NEB, B9000S)). A combination of three primary antibodies, anti-Cd11b-PE (BioLegend, 101208), anti-Cd45-APC/Cy7 (BioLegend, 103116), and anti-Cx3cr1-APC (BioLegend 149008) were diluted 1:200 in 500 uL FACS buffer and incubated with the cells at 4°C for 15 minutes. The labelled cells were washed with 5 mL PBS, pelleted at 500 *g* for 5 min, resuspended in 500 uL of FACS buffer, and finally passed through a 40 um filter (Corning, 352235). Cells were sorted on a Beckman Coulter MoFlo Astrios EQ Cell Sorter to isolate Cd11b+, Cx3cr1+, Cd45^{low} microglia.

For the microglial atlas and embryonic microglial MIA experiments, prospective S1 cortical dissections from 2-8 embryos per age per experiment were pooled, homogenized, sorted, and processed for scRNA-seq. At least two biological replicates for each age range (E12.5/13.5 [birthdate of layer 5 and 6 excitatory neurons], E14.5-E16.5 [birthdate of layer 2/3 and 4 excitatory neurons], E18.5-P1 [end of neurogenesis, transition to gliogenesis]) were obtained for the microglial atlas experiments. At least three biological replicates (pooled sets of embryos) per treatment group within each age were obtained for microglial MIA experiments. For the postnatal MIA experiments, S1 cortical blocks were dissected and pooled, homogenized, sorted, and processed for scRNA-seq as described above. Two biological replicates (pooled sets of 1 male and 1 female) were obtained for the Poly(I:C)-treated condition and four biological replicates (two pooled sets of 1 male and 1 female, one samples from an individual male and sample from an individual female) were obtained for the saline-treated condition.

Single-nucleus Suspension Preparation and FACS

Prospective S1 cortices of E15.5 embryos after MIA at E12.5 were dissected and flash-frozen in liquid nitrogen and stored at -80°C. Tail samples were simultaneously obtained from each embryo followed by DNA extraction and genotyping (see genotyping methods above) for sex and *Csf1r* genotype. Frozen tissue blocks from male *Csf1r* mutant and WT embryos were processed to obtain a single-cell suspension using papain digestion (15 minutes) (Papain dissociation kit, Worthington), following the manufacturer’s protocol. After dissociation and concentration, the pellet was resuspended in 500 uL of Nuclei Solubilization Buffer (10 mL PBS (Gibco, 15630-080) + 50 uL of 20 mg/mL BSA (NEB, B9000S) + 10 uL RNasin (Promega, N26515)) spiked with 1 uL of Hoechst and passed through a 40 um filter (Corning 352235). Nuclei were sorted on a Beckman Coulter MoFlo Astrios EQ Cell Sorter, pre-chilled to 4°C, using a 100 um nozzle, and collected into wells of a 96-well plate pre-filled with 10 uL of Nuclei Solubilization Buffer, and immediately processed for single-cell GEM formation (10x Genomics, single cell RNA sequencing 3’, Chromium v3.1). We obtained 3 biological replicates of 1-2 pooled embryos per treatment condition.

Development • Supplementary information

Table S1. Sample details and quality control information for RNA sequencing experiments

Available for download at
https://journals.biologists.com/dev/article-lookup/doi/10.1242/dev.202252#supplementary-data

Table S2. List of genes differentially expressed in cortical cell types at E15.5 after maternal immune activation, with and without microglia present

Available for download at
https://journals.biologists.com/dev/article-lookup/doi/10.1242/dev.202252#supplementary-data

Table S3. List of genes differentially expressed in microglia at E13.5 after maternal immune activation

Available for download at
https://journals.biologists.com/dev/article-lookup/doi/10.1242/dev.202252#supplementary-data

Table S4. List of genes differentially expressed in microglia at E15.5 after maternal immune activation

Available for download at
https://journals.biologists.com/dev/article-lookup/doi/10.1242/dev.202252#supplementary-data

Table S5. List of genes differentially expressed in microglia at P14 after maternal immune activation

Available for download at
https://journals.biologists.com/dev/article-lookup/doi/10.1242/dev.202252#supplementary-data

Development • Supplementary information

SUPPLEMENTARY REFERENCES

Gogos, A. *et al.* (2020) 'Sex differences in the effect of maternal immune activation on cognitive and psychosis-like behaviour in Long Evans rats', *The European Journal of Neuroscience*, 52(1), pp. 2614–2626. Available at: https://doi.org/10.1111/ejn.14671.

Kalish, B.T. *et al.* (2021) 'Maternal Immune Activation in Mice Disrupts Proteostasis in the Fetal Brain', *Nature neuroscience*, 24(2), pp. 204–213. Available at: https://doi.org/10.1038/s41593-020-00762-9.

Schindelin, J. *et al.* (2012) 'Fiji: an open-source platform for biological-image analysis', *Nature Methods*, 9(7), pp. 676–682. Available at: https://doi.org/10.1038/nmeth.2019.

Stogsdill, J.A. *et al.* (2022) 'Pyramidal neuron subtype diversity governs microglia states in the neocortex', *Nature*, 608(7924), pp. 750–756. Available at: https://doi.org/10.1038/s41586-022-05056-7.

Xuan, I.C.Y. and Hampson, D.R. (2014) 'Gender-Dependent Effects of Maternal Immune Activation on the Behavior of Mouse Offspring', *PLoS ONE*, 9(8), p. e104433. Available at: https://doi.org/10.1371/journal.pone.0104433.
