# Supplementary material for: Fetal brain response to maternal inflammation requires microglia
Source: Development. 2024 May 22;151(10):dev202252. doi: 10.1242/dev.202252 (PMC11190434; doi:10.1242/dev.202252)
Supplement: Supplementary file 1 [file develop-151-202252_review_history.pdf]

Fetal brain response to maternal inflammation requires microglia

Bridget Elaine LaMonica Ostrem, Nuria Domínguez-Iturza, Jeffrey A. Stogsdill, Tyler Faits, Kwanho Kim, Joshua Z. Levin and Paola Arlotta
DOI: 10.1242/dev.202252

Editor: Debra Silver

Review timeline

Original submission:	10 August 2023
Editorial decision:	18 September 2023
First revision received:	29 March 2024
Accepted:	9 April 2024

Original submission

First decision letter

MS ID#: DEVELOP/2023/202252

MS TITLE: Fetal brain response to maternal inflammation requires microglia

AUTHORS: Bridget Ostrem, Nuria Dominguez Iturza, Jeffrey A. Stogsdill, Tyler Faits, Kwanho Kim, Joshua Z. Levin, and Paola Arlotta

I have now received all the referees' reports on the above manuscript, and have reached a decision. The referees' comments are appended below, or you can access them online: please go to BenchPress and click on the 'Manuscripts with Decisions' queue in the Author Area.

As you will see, the referees express considerable interest in your work, but have some significant criticisms and recommend a revision of your manuscript before we can consider publication. If you are able to revise the manuscript along the lines suggested, which may involve further experiments, I will be happy receive a revised version of the manuscript. In particular the reviewers note the need for validating gene expression and microglia functional changes, as well as clarifying some findings and methods.Your revised paper will be re-reviewed by one or more of the original referees, and acceptance of your manuscript will depend on your addressing satisfactorily the reviewers' major concerns. Please also note that Development will normally permit only one round of major revision. If it would be helpful, you are welcome to contact us to discuss your revision in greater detail. Please send us a point-by-point response indicating your plans for addressing the referees' comments, and we will look over this and provide further guidance.

Please attend to all of the reviewers' comments and ensure that you clearly highlight all changes made in the revised manuscript. Please avoid using 'Tracked changes' in Word files as these are lost in PDF conversion. I should be grateful if you would also provide a point-by-point response detailing how you have dealt with the points raised by the reviewers in the 'Response to Reviewers' box. If you do not agree with any of their criticisms or suggestions please explain clearly why this is so.

Reviewer 1

Advance summary and potential significance to field

© 2024. Published by The Company of Biologists under the terms of the Creative Commons Attribution License (https://creativecommons.org/licenses/by/4.0/).

Manuscript by Ostrem et al. investigates transcriptomic changes in microglial cells following maternal immune activation in mice. Maternal immune activation is an important model of neurodevelopmental pathology associated previously with increased risk for neurodevelopmental disorders. Despite its importance, the molecular underpinnings are poorly understood.

The investigators conducted a series of MIA experiments followed by extensive and unbiased data generation using scRNAseq to illuminate microglia responses to MIA. Overall, the study represents a rich and high quality dataset representing a comprehensive overview of transcriptional landscape of cortical development under MIA conditions, and reveals long-term changes to microglia states following MIA that persist to postnatal life. These findings are extremely relevant to our understanding of the molecular underpinnings of the pathobiology induced by MIA and will lead to many hypotheses about the role of microglia in the process.

The first key finding is that microglia-depleted mice appear to be invariant to the polyI:C exposure. This finding is consistent with the observation that microglia are highly enriched for receptors of immune response mediators.

Comments for the author

The first finding that microglia depleted brain shows limited transcriptomic differences appears to be in striking contrast to the findings from a recent study conducted in brain organoids, where even in the absence of microglia interleukin 6 seems to have a very strong effect on neural stem cell proliferation (Sarieva et al. PMID: 36878967). It would be helpful to understand if the authors believe that this discrepancy is biologically meaningful, and what are the underlying underpinnings of this difference. This is in part a conceptual but also an interpretative concern that requires some form of resolution to these conflicting findings.

A second concern I have with this study is that there is a profound lack of validation. While single cell sequencing has become a cornerstone for many studies, the magnitude of changes that the investigators observe is both striking and not validated. Simple validation using in situ hybridization for key persistent differentially expressed genes following MIA within microglia would be tremendously beneficial to the rigor of this study.

Secondly, it is typically expected that studies of microglia are followed up by some form of functional analysis. It would be especially valuable to the community if the investigators, who are well equipped to conduct some validation could perform some further analysis of microglia function after MIA. This would help clarify whether the molecular changes detected using transcriptomics might have functional consequences.

Reviewer 2

Advance summary and potential significance to field

In this manuscript, Ostrem and colleagues focused on the function and molecular profile of microglia during cortical development. By using rodent maternal immune activation model and scRNA/snRNA-sequencing, the authors find that this leads to widespread gene expression changes and the cell population changes of microglia indicating the essential function of microglia cells in response to the neural inflammation. Overall, this paper provides a great resource and deep insight of understanding the heterogeneity and function of microglia.

Comments for the author

Major concerns:
1.In the manuscript line 102-103, the authors indicated CSF1R is microglia specific. Can the authors refer to other work which has validated this? If not the author should provide some data before making this statement. Such as showing this gene specific pattern in Fig1A if possible or at least perform in situ /IF on brain sections.

© 2024. Published by The Company of Biologists under the terms of the Creative Commons Attribution License (https://creativecommons.org/licenses/by/4.0/).

2.From Fig1C and FigS1C, it is difficult to see the change of abolishment of the microglia cluster as it's such a small cluster. The author should provide a value of the microglia change. It is important to know Csf1r KO is decrease microglia or totally absence. Either the percentage change number from FigS1C or the quantification of FigS1D would be insightful.
3.In Fig2E, it is difficult to see the two peak of cluster2 from this UMAP.
Especially E14.5 seems to have total fewer cells than others.

Minor concerns:
1.Images in FigS3C and FifS4E missing scale bar.
2.Where are the proliferating microglia (cluster2) located in, do they locally divide in the cortical plate or divide in germinal zone?
3.In Fig3C and Fig4A overview of the experiment, did the author only perform nonenzymatic dissociation for E13.5 and E15.5 but not P14? I understand this step is to reduce ex vivo microglial activation. Do you think it will also impact on the DEGs especially after MIA? So that means without the nonenzymatic treatment, perhaps the authors could see more genes expression changes in inflammatory microglia.

First revision

<u>Author response to reviewers' comments</u>

Dear Reviewers,

We thank the reviewers for their thoughtful comments and suggestions, which we feel have improved our manuscript considerably. Our revised manuscript is included with our re-submission, along with revised figures. We have included detailed responses to each comment below.

Comment	Response
Reviewer #1	

© 2024. Published by The Company of Biologists under the terms of the Creative Commons Attribution License (https://creativecommons.org/licenses/by/4.0/).

The first finding that microglia depleted brain shows limited transcriptomic differences appears to be in striking contrast to the findings from a recent study conducted in brain organoids, where even in the absence of microglia, interleukin 6 seems to have a very strong effect on neural stem cell proliferation (Sarieva et al. PMID: 36878967). It would be helpful to understand if the authors believe that this discrepancy is biologically meaningful, and what are the underlying underpinnings of this difference. This is in part a conceptual but also an interpretative concern that requires some form of resolution to these conflicting findings.	We agree that so far in the literature there has not been a consistently described downstream impact of microglial signaling in the setting of various infectious and inflammatory signaling. We address these issues in the discussion as follows, and now also include a citation of the suggested reference: “Microglia can play opposing, context-dependent roles in different types of brain pathology, sometimes helping promote recovery and pathogen clearance, while other times exacerbating inflammation and enabling viral replication[16,19,64,66,68]. When considering microglia as a potential future therapeutic target, it will be essential to determine whether the net impact of the microglial response in the setting of maternal inflammation is beneficial or harmful to the developing brain.”<br><br>The results of the study referenced by the reviewer likely relate to the specific compound used. The authors treated human organoids not with IL-6 alone, but with “Hyper-IL-6,” which is a soluble IL-6 receptor covalently bound to IL-6. Hyper-IL-6 can bypass the requirement for microglia and can activate a receptor that is expressed in other cell types besides microglia, IL6ST. The findings of the Sarieva et al study therefore align well with our findings and show that a microglia-derived cytokine induces widespread transcriptional changes in fetal cortical cell types.
A second concern I have with this study is that there is a profound lack of validation. While single cell sequencing has become a cornerstone for many studies, the magnitude of changes that the investigators observe is both striking and not validated. Simple validation using in situ hybridization for key persistent differentially expressed genes following MIA within microglia would be tremendously beneficial to the rigor of this study.	We appreciate the reviewer’s concerns and agree that further validation by RNA ISH would increase confidence in our findings. While validation of all findings was not feasible in this study given the large number of sequencing results, we have performed the following additional key validation experiments:<br>- Validation by RNA *in situ* hybridization of substate- defining genes for each specific embryonic microglial substates, including *Ube2c* and *Ms4a7* (previously included, now with additional images), *Spp1*, *Hmox1*, *Fabp5*, *Irf7*, which can be found in the updated Figure 2.<br>- Quantitative analysis and validation of key finding at P14 of sustained decreased *Hes1* expression after MIA as compared to saline treatment, now included in Figure 4. We performed the same analysis for *Lars2* and observed the same trend, but there was not a statistically significant decrease in *Lars2* expression after MIA by RNAscope. The analysis likely did not reach significance due to overall generally low expression levels of *Lars2* in both conditions. This experiment is also included in the updated Figure 4.

© 2024. Published by The Company of Biologists under the terms of the Creative Commons Attribution License (https://creativecommons.org/licenses/by/4.0/).

Secondly, it is typically expected that studies of microglia are followed up by some form of functional analysis. It would be especially valuable to the community if the investigators, who are well equipped to conduct some validation could perform some further analysis of microglia function after MIA. This would help clarify whether the molecular changes detected using transcriptomics might have functional consequences.	The primary goals of our study were 1) to determine whether microglia are necessary for the fetal brain response to maternal inflammation, and 2) to present a transcriptional atlas of microglial development that can serve as a reference for investigators interested in the role of microglia in development and disease. We feel that our extensive scRNAseq-based analysis and validation experiments have met these goals. We eliminated microglia from the cortex and observed that the largescale transcriptional changes seen across cortical cell types in response to MIA were nearly abolished. While further functional analysis is outside of the scope of this study, in the discussion, we cite a recent study that suggests functional consequences of the transcriptional changes that we observed specifically in microglia after MIA, such as decreased Hes1 expression at P14. In the study, Hayes at al 2022 PMID: 36171283, the authors found that microglia exposed to MIA during development were less competent to respond to immune stimuli in adulthood.<br><br>We have also included new text in the discussion section, noting the limitations of a primarily sequencing based study: “It is important to note that while RNA transcription generally correlates with protein expression, RNA sequencing may not accurately reflect simultaneous protein abundance[70].<br>Additionally, many factors, including posttranslational modifications, and mRNA and protein stability, impact when and how changes in RNA transcription affect cell function. Further studies are needed to determine the functional consequences of the transcriptional changes reported here.”
Reviewer #2	
1. In the manuscript line 102-103, the authors indicated CSF1R is microglia specific. Can the authors refer to other work which has validated this? If not, the author should provide some data before making this statement. Such as showing this gene specific pattern in Fig1A if possible or at least perform in situ /IF on brain sections.	We have confirmed in our embryonic cortical dataset that *Csf1r* is only expressed in the microglia cluster (see updated Supplemental Figure S1). We have also included additional references supporting microglia as the only known CNS cell type that expresses CSF1R (Raivich G et al J Comp Neurol 1998 PMID: 9596528, Sierra A et al Glia 2007 PMID:17203473).
2. From Fig1C and FigS1C, it is difficult to see the change of abolishment of the microglia cluster as it’s such a small cluster. The author should provide a value of the microglia change. It is important to know Csf1r KO is decrease microglia or totally absence. Either the percentage change number from FigS1C or the quantification of FigS1D would be insightful.	We have performed staining for microglia (with an antibody to IBA1) in the cortex of *Csf1r* null and control mice, which is now included, along with a quantification, in Fig. S1D. We see a 100% reduction in the number of microglia in the *Csf1r* null mice.

© 2024. Published by The Company of Biologists under the terms of the Creative Commons Attribution License (https://creativecommons.org/licenses/by/4.0/).

3. In Fig2E, it is difficult to see the two peak of cluster2 from this UMAP.<br>Especially E14.5 seems to have total fewer cells than others.	We have improved the visualization of substate proportions over time, which is now a stacked bar chart of microglial substate proportions (Fig. 2F). The two peaks of proliferating microglia are more evident in this visualization, as is the gradually increasing substate diversity reflected in the change in proportions of Ccl3+, Irf7+, Hmox1+, and Spp1+ microglia.
Minor concerns: 1. Images in FigS3C and FigS4E missing scale bar.	Thank you for noting this – scale bars added.
2. Where are the proliferating microglia (cluster2) located in, do they locally divide in the cortical plate or divide in germinal zone?	We primarily observed proliferating microglia with high *Ube2c* expression in/adjacent to the ventricular zone. However, we could also observe microglia with lower Ube2c expression further from the VZ, though none in the cortical plate. We added clarification to the text under the subheading "Embryonic microglial proliferation has two peaks." The relevant text now reads "Using RNA *in situ* hybridization, we confirmed the presence of proliferating, *Ube2c*-expressing microglia in the dorsal cortical ventricular zone, and more rarely, in the subventricular and intermediate zones (Fig. S4C). We did not observe *Ube2c*-expressing microglia in the cortical plate."
3. In Fig3C and Fig4A overview of the experiment, did the author only perform nonenzymatic dissociation for E13.5 and E15.5 but not P14? I understand this step is to reduce ex vivo microglial activation. Do you think it will also impact on the DEGs especially after MIA? So that means without the nonenzymatic treatment, perhaps the authors could see more genes expression changes in inflammatory microglia.	Nonenzymatic dissociation was performed for all microglial scRNA sequencing experiments, including at P14. We have clarified this in the methods as follows: "Live microglial suspensions from embryonic and postnatal dissected tissues were prepared for fluorescence activated cell sorting (FACS) and maintained under ice-cold conditions as previously described[48]..."

Second decision letter

MS ID#: DEVELOP/2023/202252

MS TITLE: Fetal brain response to maternal inflammation requires microglia

AUTHORS: Bridget Ostrem, Nuria Dominguez Iturza, Jeffrey A. Stogsdill, Tyler Faits, Kwanho Kim, Joshua Z. Levin, and Paola Arlotta
ARTICLE TYPE: Techniques and Resources Article

I am happy to tell you that your manuscript has been accepted for publication in Development, pending our standard ethics checks.

© 2024. Published by The Company of Biologists under the terms of the Creative Commons Attribution License (https://creativecommons.org/licenses/by/4.0/).
